# Strategic Optimization of the Flushing Operations in Lubricant Manufacturing and Packaging Facilities

**DOI:** 10.1021/acsomega.3c04668

**Published:** 2023-10-07

**Authors:** Swapana S. Jerpoth, Robert Hesketh, C. Stewart Slater, Mariano J. Savelski, Kirti M. Yenkie

**Affiliations:** Department of Chemical Engineering, Henry M. Rowan College of Engineering, Rowan University, 201 Mullica Hill Road, Glassboro, New Jersey 08028, United States

## Abstract

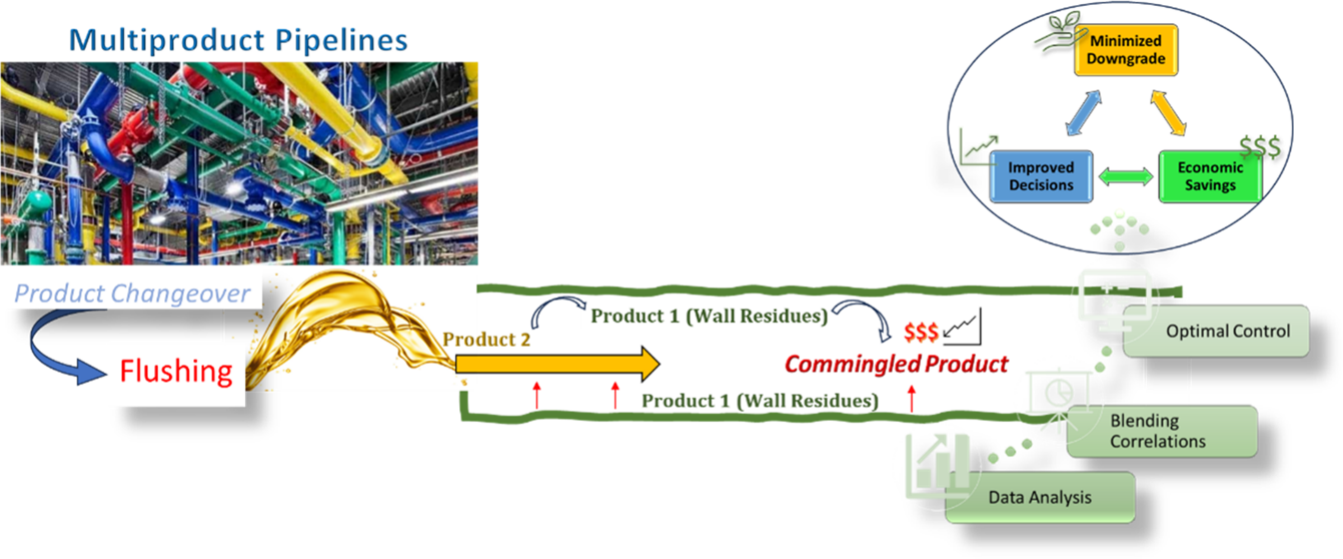

Commercial lubricant industries use a complex pipeline network for the sequential processing of thousands of unique products annually. Flushing is conducted between changeovers to ensure the integrity of each production batch. An upcoming product is used for cleaning the residues of the previous batch, resulting in the formation of a commingled/mixed oil that does not match the specifications of either of the two batches. The existing operations are based on the operator’s experience and trial and error. After a selected flush time, the samples are tested for their viscosity to determine the success of a flush. The approach results in long downtime, the generation of large commingled oil volumes, and huge economic losses. Hence, to overcome the drawback, our work introduces a solution strategy for systematically optimizing flushing operations and making more informed decisions to improve the resource-management footprint of these industries. We use the American Petroleum Institute-Technical Data Book (API-TDB) blending correlations for calculating the mixture viscosities in real-time. The blending correlations are combined with our first-principles models and validated against well-designed experimental data from the partnered lubricant facility. Next, we formulate an optimal control problem for predicting the optimum flushing times. We solve the problem using two solution techniques viz. Pontryagin’s maximum principle and discrete-time nonlinear programming. The results from both approaches are compared with well-designed experimental data, and the economic and environmental significance are discussed. The results illustrate that with the application of a discrete-time nonlinear programming solution approach, the flushing can be conducted at a customized flow rate, and the necessary flushing volume can be reduced to over 30% as compared to the trial-and-error mode of operation.

## 1 Introduction

In lubricant industries, a multiproduct pipeline system is used to process over a thousand unique products throughout the year. Different types and grades of products are manufactured and consecutively processed and packaged through sequential batch operations. The multiproduct pipelines pose many difficulties due to the high level of operational complexities.^1,2^ During a changeover operation, when switching from one product type to another, the lines must be cleaned to ensure the integrity of the new batch. The lubricant industries have many restrictions concerning product purity, and therefore, cleaning the pipelines with a foreign aqueous/nonaqueous-based solvent is prohibited as it is considered a source of contamination for the final products. Therefore, at present, these industries use a finished product from a current batch to flush the residues of the product from a previous batch. The flushing operation generates a commingled (mixed) oil that does not match the desired specifications of either of the two batches and is therefore classified as downgraded oil with a low economic value. The existing economic losses due to these drawbacks at a typical large-scale commercial facility exceed over $1M/year. Hence, this has been a long-standing and economically significant issue in these industries. To this end, our work aims to address the existing drawbacks and optimize these flushing operations by using model-predictive optimization techniques.

This article is structured into seven main sections. In the initial section, we delve into the conventional flushing operations carried out at commercial lubricant facilities, providing insights into the lubricant composition, multiproduct pipeline configurations in lubricant blending facilities, and the limitations associated with traditional flushing techniques. Section 2 sheds light on the significance of optimal control problems and their applications within the petroleum industry. Moving on to Section 3, we focus on our research methodology. Here, we delve into the recommended viscosity blending correlations from the American Petroleum Institute-Technical Data Book (API-TDB), explain our dynamic first-principles models and their validation, outline the formulation of the flushing operation as an optimal control problem, and detail the utilization of two solution techniques: Pontryagin’s maximum principle and discrete-time nonlinear programming. Section 4 is dedicated to presenting the results of our research and fostering discussions around these findings. In Section 5, we explore the economic and environmental implications of our study. Section 6 summarizes the work and hints at potential future research directions. Finally, a comprehensive list of Supporting Information for this research is provided.

### 1.1 Composition of a Lubricant

It is important to understand that a finished product used as a lubricant is a complex mixture consisting of ∼80% base stock/base oil and ∼20% functional additives. The base stock contributes significantly to the finished product properties, whereas the additives are chemicals that are added to enhance the existing properties or impart desired properties to the base stock.^3^ The production of finished lubricants from base stocks and functional additives is commonly referred to as oil blending since it primarily involves a mixing process without any chemical reactions.^4^ Lubricants have numerous applications in a variety of fields, and depending upon their final use, they can be classified into different families such as (i) engine oils (petroleum and diesel engines, aircraft, marine engines), (ii) turbine oils, (iii) gear oils, (iv) quench oils in metalworking, (v) insulating oils, (vi) chain lubricants, and (vii) hydraulic oils. Each of these oil types possesses distinct characteristics tailored to meet specific system requirements. Lubricants are complex fluids that fulfill a range of protective and functional roles, such as creating a hydrodynamic film between moving parts, dispersing heat, trapping contaminants, neutralizing acids, and preventing corrosion. The efficient operation of a modern lubricant blending facility plays a crucial role in ensuring the precise delivery of high-quality lubricants with optimal performance to customers.

### 1.2 Pipeline Configuration and Existing Flushing Operations at Lubricant Blending Plants

In a generic lubricant facility, the configuration of the production system is notably complex. It starts at the feed tanks in the tank farm, proceeds into the blending room, where distinct product formulations are created by combining base stocks with functional additives in blending vessels, and ultimately extends to the packaging station, where the final products are packaged in various styles such as bottles, pails, and drums. Each type of lubricant oil possesses unique characteristics, necessitating their segregation to maintain the highest quality standards.^5,6^ Hence, the system undergoes cleaning procedures between changeover operations to prevent any cross-contamination. The processing pipelines encompass straight sections joined with different bends (typically 45 and 90°), turns, and ancillary equipment like micron-sized filters. For the cleaning of the straight sections of these pipelines, a method known as “pigging” is employed. This method uses a polymeric device called a “pig”,^7^ which got its name due to the distinctive squealing sound it generates as it travels through the pipelines. Over time, the term “pig” evolved into an acronym, standing for “pipeline inspection gauge.” The pig functions as a scraping device propelled through the pipelines by compressed air. It creates a seal against the inner walls of the pipes and effectively removes accumulated oil deposits. However, a significant limitation arises from the rigidity of these polymeric pigs, preventing them from navigating through variable diameter pipelines, negotiating 90° bends, handling turns, or dealing with ancillary equipment like filters. As a solution to clean the remaining sections of the system, a method involving the use of a flushing oil is employed. This flushing oil is a finished product from an upcoming batch that is utilized to cleanse any residues of the preceding batch. This practice results in the creation of a mixed or commingled oil that does not adhere to the specifications of either of the two batches, and as a consequence, it is classified as a downgraded product with low economic value.

### 1.3 Viscosity: The Paramount Factor Affecting Lubricant Integrity

Viscosity is considered one of the most important factors influencing the lubricating properties of an oil.^8,9^ When a lubricant’s viscosity exceeds the optimal range, it can lead to flow-related issues, giving rise to problems like heightened friction, increased heat generation, elevated wear and tear, and difficulties in starting in colder conditions. Conversely, when a lubricant’s viscosity falls below the desired level, it may fail to adequately coat and safeguard the components, as intended. This can result in detrimental outcomes, including excessive wear, heightened friction and heat, heightened vulnerability, and greater susceptibility to contamination. Hence, maintaining precise viscosity levels during the manufacturing process is crucial to ensuring the ultimate quality of the lubricant product.

### 1.4 Challenges with the Traditional Flushing Operations and Quality Control Techniques

Currently, the flushing procedures primarily rely on a trial-and-error approach, which is regulated by a flush timer. An operator selects a flush duration based on their previous experience with a particular product. At the conclusion of the flushing process, samples are collected and sent to the laboratory, where they undergo a series of physical and chemical tests to confirm the lubricant’s top-grade quality. Common physical tests encompass measurements of viscosity, specific gravity, and color, while typical chemical tests include assessments of flash and fire points. Out of the several tests, viscosity is the preliminary and most crucial test that ensures the batch quality and success of the flush. If the viscosity test results fall outside the desired range, additional flushing is performed, and this cycle continues until the desired specifications are met. In the traditional method of flushing, sampling serves as the primary technique for quality control and monitoring. However, this sampling approach results in extended hold times and downtime. Furthermore, in many instances, it leads to excessive flushing, generating large quantities of commingled oil, and causing significant economic losses for these industries. With this objective in mind, our study aims to tackle these limitations and investigate alternative operational methods with the goal of reducing the commingled oil volumes and enhancing the economic and resource-management footprint of flushing operations in the lubricant industry.

### 1.5 Predictive Modeling Approaches for Enhancing Flushing Operations

In recent years, optimization techniques have gained a growing interest in the petroleum industry. Mixed-integer linear programming (MILP) and mixed-integer nonlinear programming (MINLP) models have been widely used for developing systematic scheduling plans and calculating commingled oil volumes for multiproduct pipelines.^10−16^ However, scheduling plans are not sufficient for eliminating the generation of commingled products. Hence, over the years, researchers have reported that the extent of mixing depends on various factors including fluid properties, operating conditions, and flow regimes.^17−20^ Major oil companies are developing models for calculating the commingled oil volumes.^21−23^ These models are based on empirical correlations that are applicable only to their respective pipeline networks. There is no widely accepted correlation that can be used in all actual scenarios.^24^

Extensive studies have been conducted on the formation of commingled oil within crude and refined petroleum pipelines, which transport multiple fluids consecutively during continuous operations. Table 1 provides an overview of important features from recent publications by various researchers.

**Table 1:** Previous Studies on Multiproduct Petroleum Pipelines

author	feature
Liu et al. (2020)^21^	studied the formation mechanism of mixed oil at inclining pipeline sections and developed an empirical correlation for calculating the volume of mixed oil formed
Liao et al. (2019)^25^	developed a MILP continuous-time formulation for the detailed scheduling of a branched pipeline system with a single refinery and multiple depots. The proposed work allows multiple batches to be processed by a node over a single slot
Liu et al. (2019)^26^	studied the influence of dead-legs on the formation of mixed oil. The authors reported that the mixed oil formation rate in the dead-leg is exponentially related to the flow speed
He et al. (2018)^24^	developed a numerical model to simulate the mixed oil segment transported in a crude oil pipeline. The authors used several dimensionless indices including mixed segment length, axial tailing length, and radial difference to measure the concentration distribution in the mixed segment
Maleki and Frigaard (2016)^27^	analyzed the turbulent flows of shear-thinning fluids in pipe and channel geometries. The authors reported the relationship between the mean velocity and the wall shear stress and proposed the necessity of including an analysis of wall layers in studying dispersion
Patrachari and Johannes (2012)^17^	determined the extent of mixing by using different fluid properties and an axial dispersion coefficient. The author proposed a model that incorporated turbulent and viscous effects in predicting the interfacial contamination volume
Rejowski and Pinto (2008)^28^	proposed a novel MINLP formulation based on a continuous time representation for the scheduling of multiproduct pipeline systems. The influence of the number of time intervals representing the transfer operations is studied and several configurations for the booster stations are tested

However, to the best of the authors’ knowledge, no prior studies have been published concerning multiproduct pipelines utilized in the production and packaging of finished lubricants. Given this context, drawing upon these foundational concepts and the existing body of literature, we conceived the idea of employing optimization techniques and framing the flushing operation within the lubricant industry as an optimal control problem. Our developed models underwent validation through systematically designed and executed plant experiments conducted at our collaborative facility, which ranks among the global leaders in the production of finished lubricant products.

## 2 Optimal Control Problems and Applications

The flushing operation involves controlling a dynamic system, i.e., the system that evolves over time. Hence, this work employs the use of optimal control theory, which is a branch of mathematics that finds optimal ways to control dynamic systems.^29,30^ Previously, control methods have been successfully applied in the petroleum industry for enhancing working systems. Ramirez^31^ studied the application of optimal control theory for enhancing oil recovery techniques. Sarma et al.^32^ implemented the adjoint solution technique in optimal control to optimize the production techniques in oilfields. Sarma et al.^33^ implemented optimal control for real-time production optimization and reservoir management. Zhang et al.^34^ used a gradient-based optimal control approach for maximizing the profits in oil production. Hasan^35^ used the adjoint method and the line search method in optimal control for maximizing the oil revenue in petroleum reservoir systems. Yang et al.^36^ used optimal control strategies for optimizing the produced water treatment systems. With reference to the existing literature, this work uses the optimal control solution strategy for solving the developed pipeline flushing problem for lubricant industries.

Optimal control deals with the properties of control functions such that these functions, when inserted in differential equations, give a solution that minimizes or maximizes a performance index. In engineering applications, the control function is a control strategy. The differential equations describe the dynamic response of the mechanism to be controlled and depend on the control strategy employed.^37^ The evaluation of the time-dependent operating profiles, in terms of the control variable, is used for optimizing the process performance.^38^ Due to the dynamic nature of the decision variables, optimal control problems pose much more complexities than other optimization problems where the decision variables are scalar.

In this work, the dynamic system under study is the pipeline flushing operation at the lubricant facility. The way of controlling the state of our system is through the flow rate of the oil that is used for flushing. Hence, our control variable is the flushing oil flow rate. We achieve the theoretical optimal flow rate profile and report valuable information for designing and controlling the flushing operation. The purpose is to find a flow rate control policy that can change with time by using dynamic optimization. The optimal control problems are solved using mathematical principles, including dynamic programming, calculus of variations, discretized nonlinear programming, and Pontryagin’s maximum principle.^39,40^ Calculus of variations and dynamic programming involve second-order differential equations and partial differential equations, which lead to mathematical complexities. In contrast, Pontryagin’s maximum principle entails only first-order differential equations, which reduces the mathematical and computational complexities and makes this method more attractive than the other two.^38^

## 3 Materials and Methods

The materials and methods are divided into four subsections. Section 3.1 discusses the viscosity blending correlations and their validation for lubricant mixtures. Section 3.2 explains our first-principles models for flushing operations in multiproduct lubricant pipelines. Section 3.3 describes the validation of the models through well-designed experimental data at the partnered lubricant facility. Section 3.4 illustrates the development of the optimal control problem and the solution methodology using Pontryagin’s maximum principle and the discrete-time nonlinear programming (NLP) solution approach.

### 3.1 Viscosity Blending Correlations

The conventional methods of testing viscosity as per the ASTM D445 standards^41^ require a long-running time (approximately 20–30 min), leading to extended operational downtime. Thus, this work addresses the existing drawback and enables better in-line controllability of the flushing operation by developing models for predicting the viscosity of the lubricant mixture/blend in real time. The viscosity blending correlations are combined with the component balance equations for the multiproduct pipeline systems.

Our work uses the viscosity blending correlations recommended by the American Petroleum Institute’s Technical Data Book (API-TDB).^42,43^ The correlation (represented by eq 1) calculates the viscosity of the blend of two or more components as the cubic-root average of the individual component viscosities. Furthermore, this gives us an understanding of the concentration of the individual components of the blend. Through this equation, we will predict during a flush how the mixture viscosity attains the desired specifications of the new/upcoming lubricant with every time step.

The suffix “A” stands for residual lubricant, and the suffix “B” stands for upcoming lubricant (flushing lubricant).
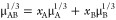
1Here, μ_AB_ is the viscosity of the mixture of lubricants A and B (cSt), μ_A_ is the viscosity of the residual lubricant (cSt), μ_B_ is the viscosity of the upcoming lubricant (flushing oil) (cSt), *x*_A_ is the mass fraction of the residual lubricant, and *x*_B_ is the mass fraction of the upcoming lubricant (flushing oil).

Note: In ref (31), *x*_A_ and *x*_B_ in eq 1 stand for mole fractions of the pure components. However, in this work, we noticed that the mole fractions and mass fractions for the lubricant mixtures had a negligible difference, and therefore, to eliminate computational complexities, we considered *x*_A_ and *x*_B_ to be the mass fractions.

To validate the API-TDB-recommended blending correlation for lubricant mixtures, a series of experiments were conducted following this stepwise procedure.

Step 1: We prepared known compositions of lubricant mixtures, ranging from a volume fraction of 0.1–0.9, with each sample incrementing by 0.1. This resulted in a total of 11 samples. We ensured the accuracy of sample preparation by selecting a total sample volume of 25 mL and carefully measuring the weights while mixing the two lube oils. This allowed us to obtain precise mass fraction data for each of the 11 samples.

Step 2: The kinematic viscosity of each sample was determined according to the ASTM D445 guidelines,^41^ using Ubbelohde viscometers and a constant-temperature bath setup.

**Figure 1:**
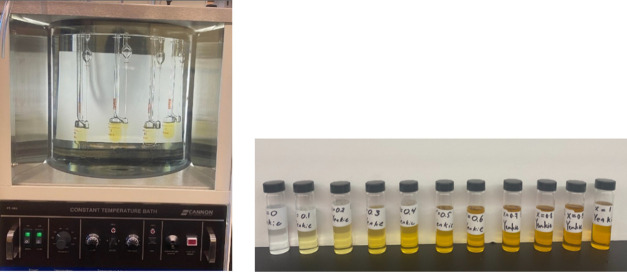
Constant-temperature bath setup with Ubbelohde viscometers for kinematic viscosity measurement (left). Sample vials of known mass fractions of lubricant mixtures (right).

**Figure 2:**
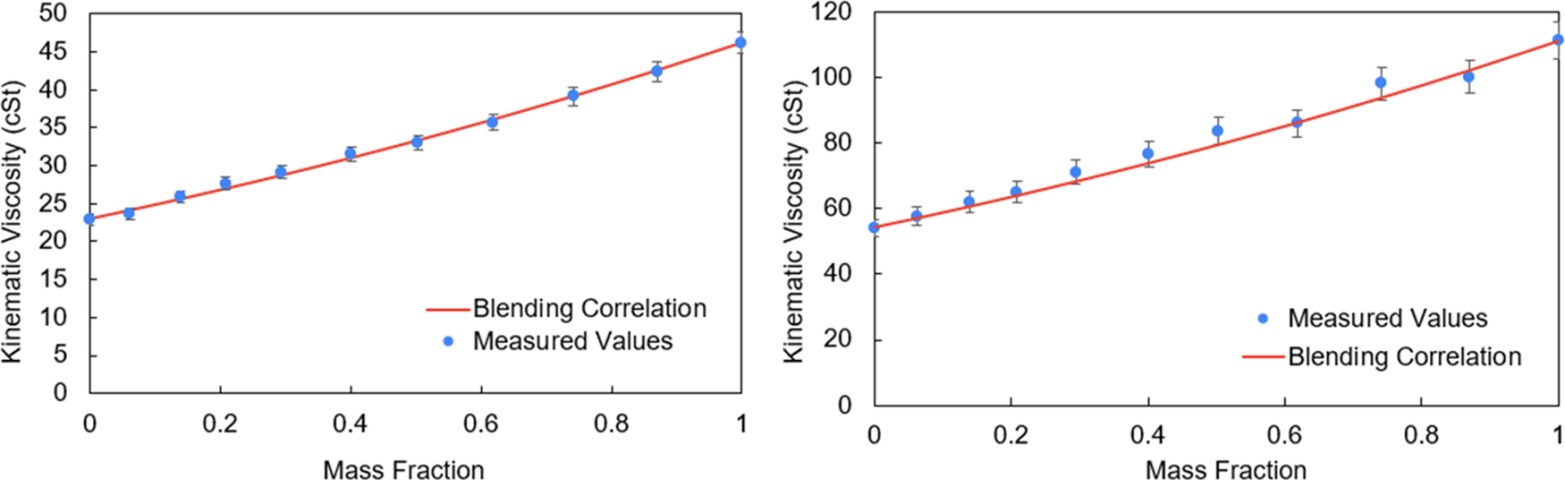
Validation of the API-TDB blending correlation for lubricant mixtures. Blue data points indicate the experimentally measured values of kinematic viscosity, and the red curve illustrates the calculated value from the blending correlation.

Step 3: We compared the experimentally measured viscosity values with those calculated using the blending correlation. In Figure 1 (left), one can see the Ubbelohde viscometers and the constant-temperature bath setup used in the experiments. The changing colors depicted in Figure 1 (right) visually represent the increasing concentration of the golden-colored lube oil in a mixture of translucent and golden lube oil with known mass fractions. We conducted the validation of calculated viscosity values against experimentally measured values for two distinct lubricant mixtures, as illustrated in Figure 2. The agreement within a 5% margin of error confirmed the applicability of the blending correlation to lubricant mixtures.

Figure 1 (left) illustrates the Ubbelohde viscometers and a constant-temperature bath setup that was used for the experiments. The changing colors in Figure 1 (right) visually depict the increasing concentration of the golden-colored lube oil in a mixture of translucent and golden lube oil of known mass fractions. The validation of the calculated viscosity values against the experimentally measured values was carried out for two distinct lubricant mixtures, as shown in Figure 2. The agreement within a 5% margin of error confirmed the applicability of the blending correlation to lubricant mixtures.

### 3.2 Dynamic First-Principles Model of Flushing Operation

**Figure 3:**
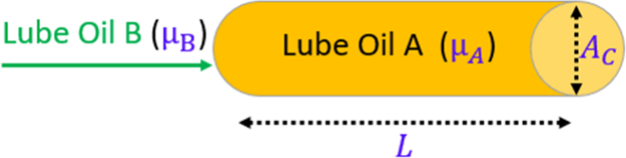
Illustration of a changeover operation in a lubricant pipeline with a cross-sectional area “*A*_C_” and total length “*L*”.

Let us consider that initially, lubricant A is processed through the pipelines. After the packaging of “lubricant A” is completed, the upcoming batch of lubricant B is to be packaged as illustrated in Figure 3. Hence, the pipelines must be flushed with lubricant B until the desired specifications are reached.

The list of model parameters and variables used for the problem formulation is as follows.

Model parameters: μ_A_, viscosity of the residual lubricant (cSt); μ_B_, velocity of the upcoming lubricant (flushing oil) (cSt); ρ_A_, density of lubricant A (kg/m^3^); ρ_B_, density of lubricant B (kg/m^3^); *A*_C_, cross-sectional area of the pipeline (m^2^); *L*, total length of the pipeline (m); and *V*, total volume of the pipeline (m^3^).

Model variables: μ_AB_t__, viscosity of the blend of lubricants A and B (cSt); *m*_A_t__, mass of lubricant A (kg); *m*, total mass of the system (kg); *x*_A_t__, mass fraction of lubricant A; *x*_B_t__, mass fraction of lubricant B; *Q*_t_, volumetric flow rate of lubricant B (m^3^/s); and *t*, flushing time (s).

A general mass balance equation for lubricants A and B is derived as follows:
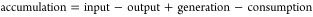
2

Assumptions: (1) Initially, the pipeline is completely filled with lubricant A before lubricant B is processed. (2) The densities of lubricants A and B are approximately the same. (3) There is no chemical reaction taking place in the pipeline.

The model parameters ρ_A_, ρ_B_, *A*_C_, *L*, and *V* remain unchanged for the system. However, the parameters μ_*A*_ and μ_*B*_ change with respect to each case study and the effect of the changes have been discussed in detail in Section 4 of the paper.

The generation and consumption term in eq 2 can be neglected as there is no chemical reaction taking place in the pipeline. Therefore,
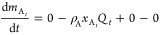
3Writing eq 3 in terms of the mass fraction
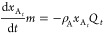
4
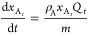
5
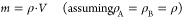
6
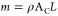
7Substituting eq 6 in eq 5
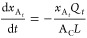
8Similarly,
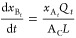
9Differentiating eq 1 w.r.t “*t*” and substituting the values of and 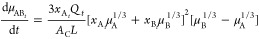
10Equations 8–10 represent our first-principles models. These models were then validated against well-designed experimental data. More details with regard to the validation are discussed in Section 3.3.

### 3.3 Validation of First-Principles Models

Our partnered lubricant facility processes over 15 000 unique products in a given production year. We first started by collecting and analyzing data from the regular flushing operations conducted by this facility. Our analysis gave us a strong indication that the flushing operation was not optimum, and the flush time was chosen based on the operator’s experience and through the trial-and-error method. Therefore, in the subsequent phase, we formulated and executed a set of structured experiments to acquire empirical data points for the purpose of validating our mathematical models. The procedural sequence observed during these experiments within the plant is outlined as follows.

Step 1: 25 mL sample bottles were prepared, ensuring proper labeling.

Step 2: Prior to the flush, the initial volume of the feed tank was recorded.

Step 3: The flush procedure was initiated with a well-defined time.

Step 4: Over a flush duration spanning from 60 to 120 s, based on the operator’s experience, samples were collected at every 10 s interval.

Step 5: Upon completion of the flush, the timer and sample collection were halted.

Step 6: The final volume of the feed tank was then documented.

Step 7: Subsequently, the collected samples were dispatched to the laboratory for kinematic viscosity testing.

**Figure 4:**
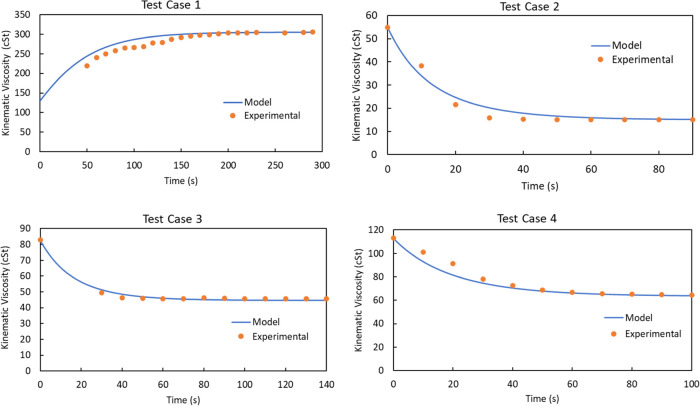
Validation of the developed first-principles models with empirical data points collected from well-structured experiments conducted at the partnered lubricant facility.

The samples taken at 10 s intervals provided us with valuable insights into how the viscosity of the mixture, comprising residual and fresh lubricant, progressed toward meeting the desired specifications of the new lubricant at each time increment. The flushing data for 25 different changeover operations were analyzed and compared against the mathematical models. Confirmation of the accuracy of our models in representing the flushing operation was established with agreement within a 7% margin of error. Figure 4 provides a comparative analysis between the experimental data points and the simulated outcomes for four specific changeover operations. We have singled out these four changeovers from the total of 25 as they offer a clear and illustrative depiction of the transition, providing further insight into the effectiveness of our models in capturing these transition scenarios. Table 2 illustrates the viscosity gradient between the residual and flushing oil and the flushing flow rates for each of these test cases.

**Table 2:** Viscosity Gradient between the Residual and the Flushing Lubricant, and Flushing Flow Rate of the Selected Test Cases

test case	viscosity of residual oil (cSt)	viscosity of flushing oil (cSt)	viscosity gradient (cSt)	flushing flow rate (m^3^/s)
1	130.26	305.39	175.13	0.003
2	54.85	15.06	39.79	0.006
3	82.84	44.8	38.04	0.006
4	113	63.2	49.8	0.005

### 3.4 Formulation of the Optimal Control Problem

Our objective of the epoch (lubricant B) is to make the upcoming oil (lubricant B) completely free of the residual oil (lubricant A) at the final collection point. Hence, the viscosity of the blend at the final time point should be equal to the viscosity of lubricant B. Mathematically, our objective can be formulated to minimize the difference between the viscosity of the blend and the viscosity of lubricant B by finding an optimum flushing time, as shown in eq 11.
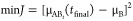
11

The state of our system is controlled through the flow rate of lubricant B (flushing oil). Hence, the variable *Q*_*t*_ represents the control variable of the system. The process performance is determined by attaining the desired viscosity of lubricant B. Given the values of the state variables *x*_*i*_ [where x_*i*_ = (*x*_A_*t*__, *x*_B_*t*__, μ_AB_*t*__)] and the control variable *Q*_*t*_ at time *t*, the differential equations eqs 8–10 specify the instantaneous rate of change in the state variables. The developed optimal control problem was solved using two solution approaches, viz. Pontryagin’s maximum principle and discrete-time nonlinear programming (NLP).

#### 3.4.1 Method#1: Pontryagin’s Maximum Principle

The application of the maximum principle requires the introduction of additional variables known as adjoint variables and a Hamiltonian. Three adjoint variables “*z*_*i*_“, corresponding to each of the state variables, and a Hamiltonian were used in this work. The introduced adjoints must satisfy eq 13, and the Hamiltonian must satisfy eq 14. The details of the derived adjoint and Hamiltonian equations are provided in Sections S3.2 and S3.3. Table 3 summarizes the various quantities that describe our model.
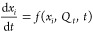
12
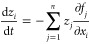
13
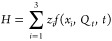
14

**Table 3:** Quantities that Describe the Developed First-Principles Mathematical Model

quantity	mathematical model
parameters	μ_A_, μ_B_, *A*_C_, *L*
state variables	*x*_i_ = [*x*_A_t__, *x*_B_t__, μ_AB_t__]
state equations	d*x*_i_/d*t* = *f*(*x*_i_, *Q*_t_, *t*)
adjoint equations	d*z*_i_/d*t* = −∑_j = 1_^n^z_j_∂_f_j__/∂_x_i__
Hamiltonian equations	*H* = ∑_i = 1_^3^z_i_* f*(*x*_i_, *Q*_t_, *t*)

The system results in a two-point boundary value problem since we have initial conditions for the state variables and final conditions for the adjoint variables. The initial conditions for the state variables are *x*_*i*_ (*t*_0_)= [1 0 μ_A_], and the final conditions for the adjoint variables are *z*_*i*_ (*t*_f_)=[ 0 0 –1 ]. Furthermore, the total flush time ranges between 60 and 290 s.

For evaluating the Hamiltonian derivative, we use an analytical method proposed by Benavides and Diwekar, which introduces an additional variable corresponding to each state variable and adjoint variable. The variable θ_*i*_ corresponds to each of the state variables *x*_*i*_ and the variable Φ_*i*_ corresponds to each of the adjoint variables *z*_*i*_, respectively (described in detail in Section S1).
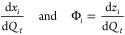
15
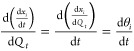
16
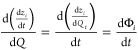
17
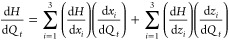
18Thus, the complete model will consist of three state equations (eqs 8–10), three adjoint equations, and 12 Hamiltonian equations.

**Figure 5:**
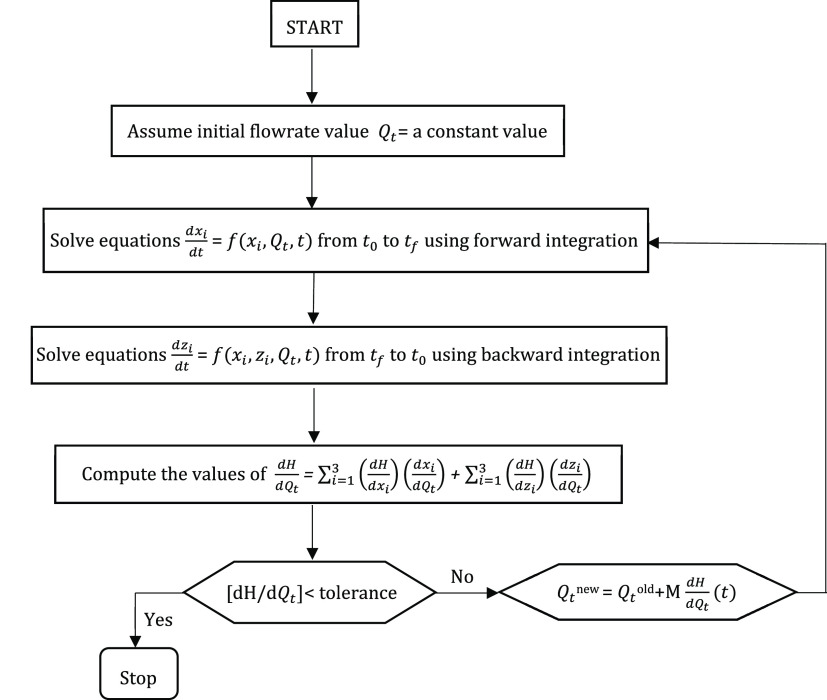
Flowchart of the solution technique using the maximum principle approach.

The algorithm starts with the initial guess of the flow rate *Q*_*t*_. Next, state equations represented by eq 12 are solved for the interval of *t*_0_ to *t*_*f*_ using forward integration and employing Euler’s method. Then, the adjoining equations represented by eq 13 are solved using backward integration. Next, the optimal control variable *Q*_*t*_ is obtained by finding the extremum of the Hamiltonian at each time step, using the optimality condition of [|d*H*/d*Q*_*t*_|] < tolerance. Our tolerance limit is zero. If the optimality condition is not satisfied, the flow rate *Q*_*t*_ is updated using the gradient, such that the updated flow rate profile improves the objective function. Figure 5 shows the flowchart for the solution approach.

In this work, we experienced that the execution time for Pontryagin’s maximum principle algorithm exceeded over 60 000 s. Hence, to overcome this drawback, we used the discrete-time nonlinear programming (NLP) solution method. In the discrete-time NLP solution approach, the total time is discretized into “*n*” known intervals, and the state equations are solved for each interval. More details of the method are discussed in the following section.

#### 3.4.2 Method#2: Discrete-Time Nonlinear Programming (NLP) Solution Approach

**Figure 6:**
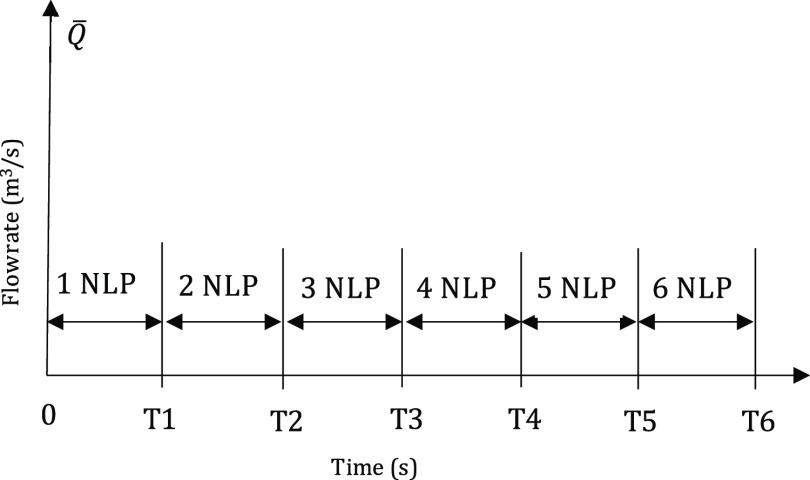
Discretization of total time into equal intervals to solve the state equations within each interval.

In the discrete-time NLP solution method, the total flush time is discretized into known “*n*” equal intervals. The objective function stays the same, and it is subjected to the integrated form of the state equations [eqs 20 and 21]. These equations are solved for each interval. Let us consider for example the total flush time was 60 s and we divide the total flush time into six equal intervals of 10 s each as illustrated in Figure 6. The solution algorithm solves the state equations for each interval. In this solution approach, the control variable “*Q̅*” is provided as a vector and we specify the system-specific maximum and minimum constraints for our control variable (flow rate of lubricant B).
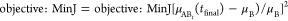
19

Subject to
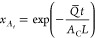
20
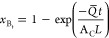
21Equation for μ_AB_*t*__
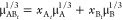
22

**Figure 7:**
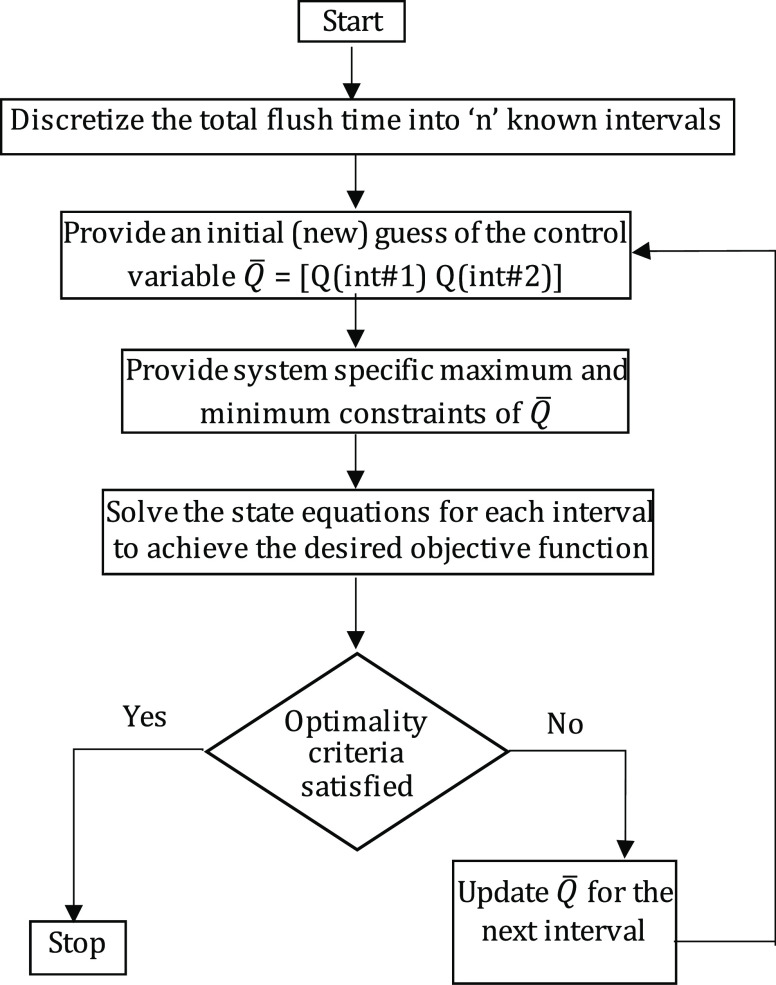
Flowchart of the solution technique using the discrete-time nonlinear programming (NLP) solution approach.

Next, we solve the state equations for the first interval to achieve the desired objective function. If the optimality criteria are not satisfied, the flow rate is updated for the next interval such that the updated flow rate profile improves the objective function. The iterations continue for “*n*” intervals until the desired optimality condition is achieved, i.e., the difference in the viscosities of the blend and the viscosity of lubricant B is minimized. In other words, the desired specifications of the new lubricant B is reached. The choice of our decision variable is based on time because in real-world scenarios, the plant operators at these facilities can provide the input in terms of time. Therefore, we study the optimum flow rate in a given time interval to conduct a successful flush. The developed discrete-time NLP problem is solved in MATLAB using the constrained optimization algorithm “fmincon”. Figure 7 depicts the flowchart of the solution approach.

## 4 Results and Discussions

The results and discussions are divided into two subsections. Section 4.1 illustrates the maximum principle solution approach. Section 4.2 explains the comparison of maximum principle solution results with the discrete-time NLP results and its comparison with the experimental data.

### 4.1 Pontryagin’s Maximum Principle Solution Method

**Figure 8:**
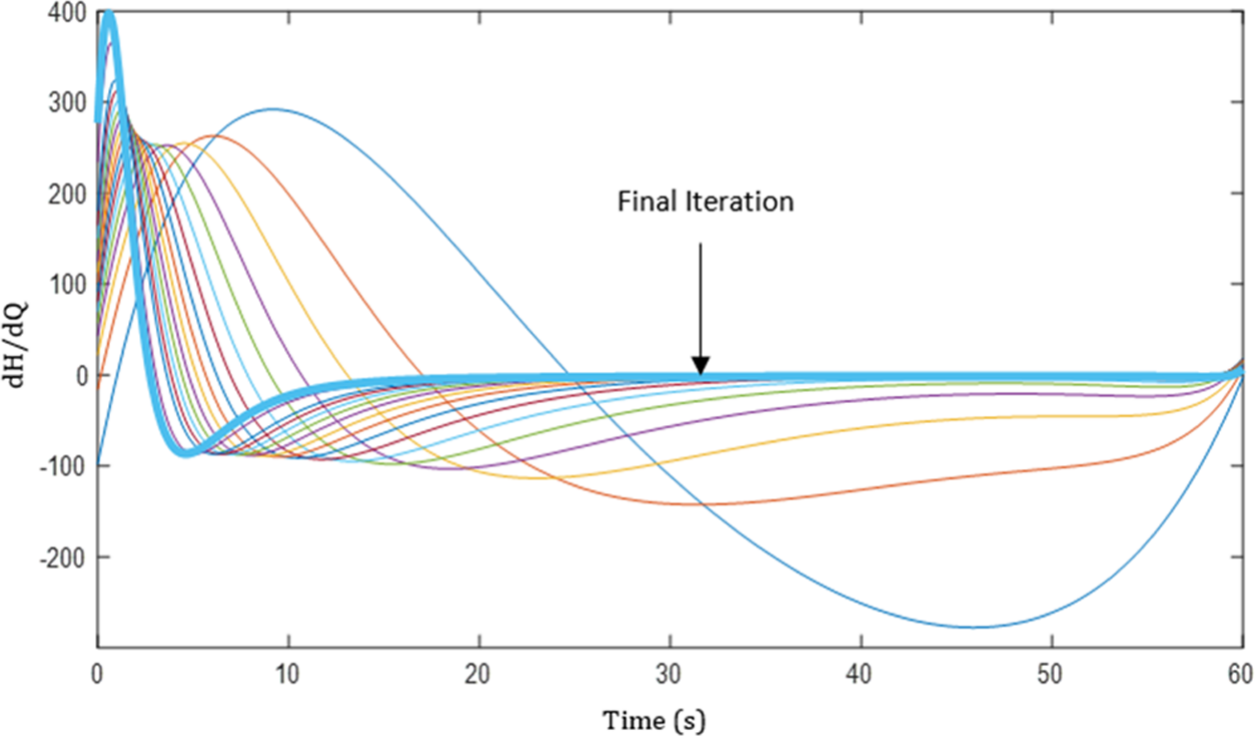
Graph depicting the Hamiltonian gradient profiles for each iteration, showcasing a progressive reduction in value with each step and ultimately reaching the tolerance limit in the final iteration.

**Figure 9:**
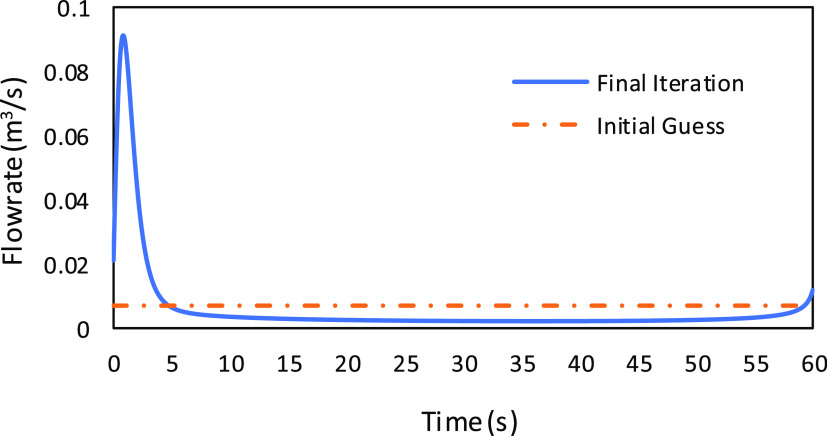
Profile of the flow rate corresponding to the final iteration where the Hamiltonian gradient achieves the desired tolerance limit.

The derivative of Hamiltonian profiles at different iterations for a case study is shown in Figure 8. It can be observed that the d*H*/d*Q*_*t*_ value decreases with every iteration. The final iteration value lies within the given tolerance limit; hence, we conclude the flow rate to be optimal, and the corresponding profile is shown in Figure 9.

The final iteration of the optimal flow rate profile was used for simulating the state equations. The goal was to predict the time step at which the viscosity of the blend reaches the desired viscosity limits of lubricant B. The comparison between the optimum flushing time predictions via the maximum principle solution approach, the discrete-time NLP solution approach, and the experimental data is discussed in Section 4.2.

### 4.2 Comparison of Different Solution Methods

**Figure 10:**
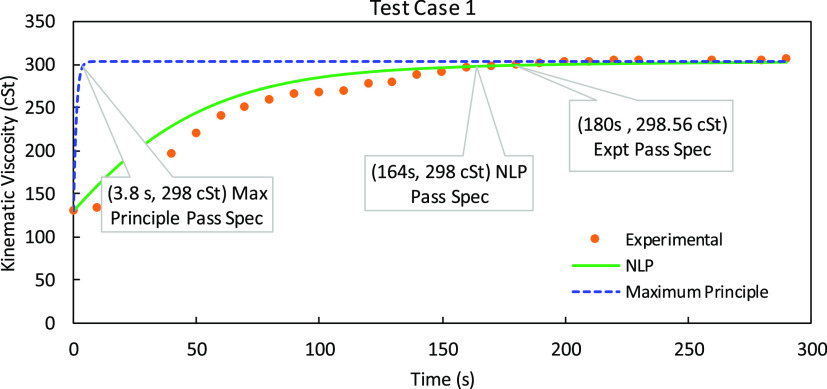
Comparison of maximum principle and discrete-time nonlinear programming (NLP) solution approach for Test Case 1 where the viscosity of the residual lubricant is 130.26 cSt and the desired viscosity of the new lubricant is 298.56 cSt.

In Figure 10, we show a comparison of the maximum principle and the discrete-time NLP solution approach for test case 1.

**Figure 11:**
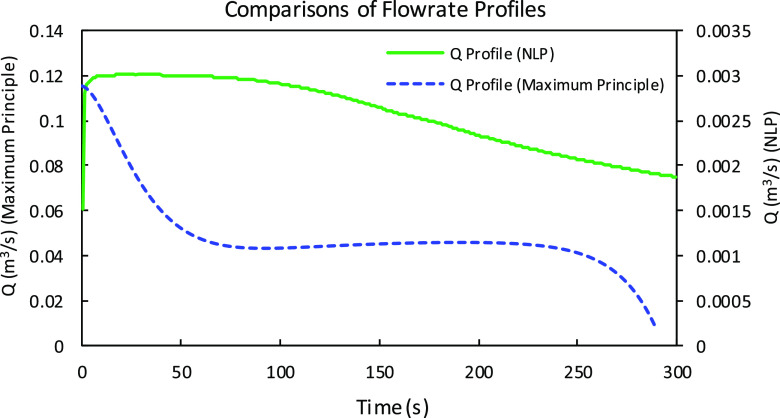
Comparison of the optimal flow rate profiles for the maximum principle and the discrete-time nonlinear programming (NLP) solution methods for Test Case 1.

**Figure 12:**
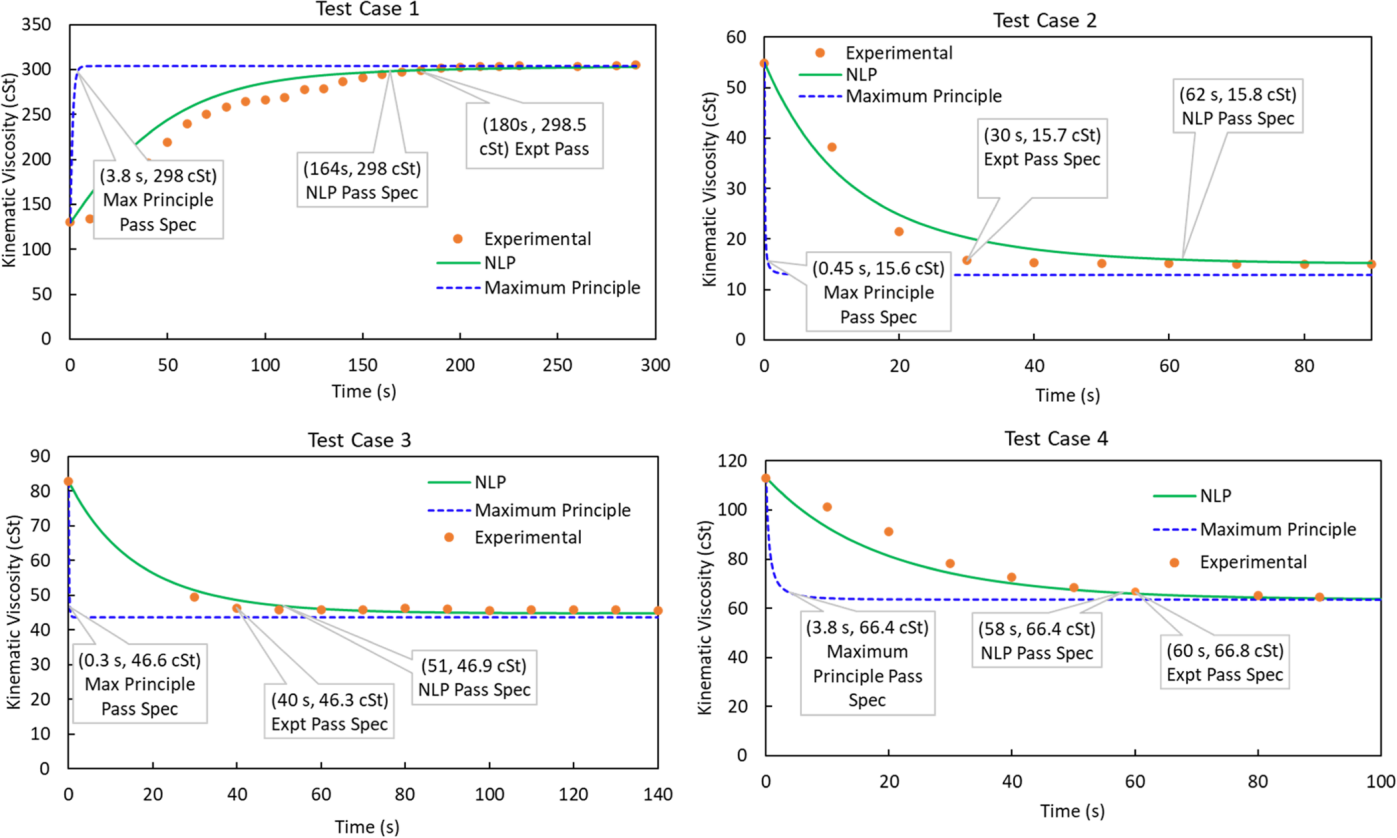
Comparison of maximum principle and the discrete-time NLP solution approach.

We plot the flush time against the kinematic viscosity of the collected lubricant blend samples. Test Case 1 was a changeover operation where the viscosity of the residual lubricant was 130.26 cSt and the desired viscosity of the new lubricant was 305.39 cSt. According to industrial standards, flushing can be stopped when the sample reaches a value within 5% of the desired value. At the partnered industrial facility, the operator, based upon his experience with this specific product, chose a total flush time of 290 s. However, as explained in Section 3.3, for our designed experiments conducted at this facility, we collected samples at an interval of every 10 s for the total flush time. We tested these samples for their kinematic viscosity, and the experimental data points are shown by the orange scattered plot in Figure 10. As observed, the desired passing specification of 298.56 cSt was achieved right at the 18th sample corresponding to the total flush time of 180 s. Our results from the discrete-time NLP solution method (represented by the smooth green curve in Figure 10) show that if the operation was to be conducted at an optimal flow rate profile the desired specifications would be achieved at a total flushing time of 164 s. The execution time for the problem was 250 s. Furthermore, we ran the simulation for Pontryagin’s maximum principle solution approach (illustrated by the dashed curve in blue in Figure 10). The execution time for the problem was 70,360 s, and the results indicate that the desired specification will be achieved at 3.8 s of the flushing time. However, this is not possible in a practical scenario. We further compared the optimal flow rate profile for the nonlinear programming solution method and the maximum principle solution method shown in Figure 11. The flow rate profile for the maximum principle method (dashed curve in blue in Figure 11) starts with the initial guess and starts decreasing for the next 50 s of interval. It stays constant until 250 s and further starts decreasing until the last interval. The optimal flow rate profile for the NLP solution method starts with the initial guess, reaches a maximum value, and slightly decreases for the next few intervals until the end time. Similarly, we compare the results for the change in kinematic viscosity against time for 25 changeover operations and compare the results with the experimental data. The graphs for the four changeovers are shown in Figure 12. For Test Case 2, the NLP prediction for the optimum flush time was almost double as compared to the experimental data points observed via the 10 s interval sample collection. However, the actual flush time chosen by the operator was 90 s, which was significantly higher than the necessary flush required. Furthermore, the maximum principle prediction for the optimum flush time was only a fraction of a second. For Test Case 3 and Test Case 4, the NLP results obeyed very closely with the experimental data. Furthermore, the optimum flush time for the maximum principle was still below 5 s. The execution time for the NLP solution method was within 20 s, whereas the execution time for the maximum principle exceeded over 70 000 s.

## 5 Economic and Environmental Significance

**Figure 13:**
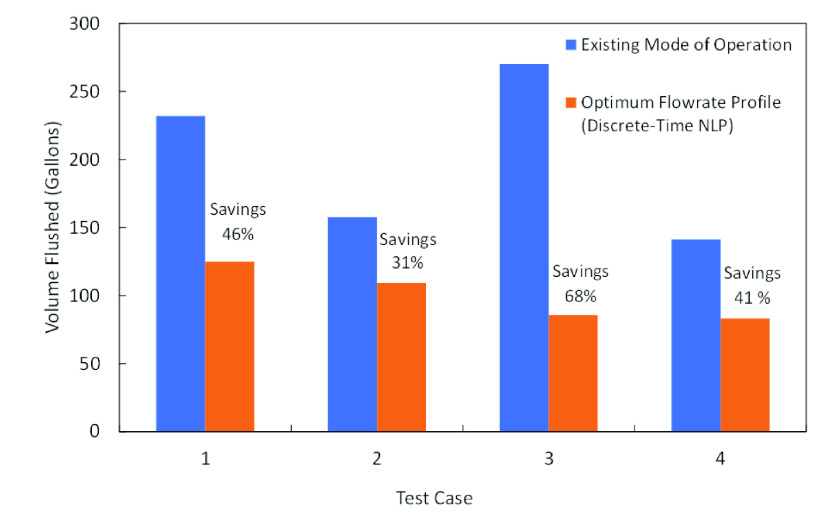
Comparison of the necessary flushing volume in the existing mode of operation and the discrete-time NLP solution approach.

**Figure 14:**
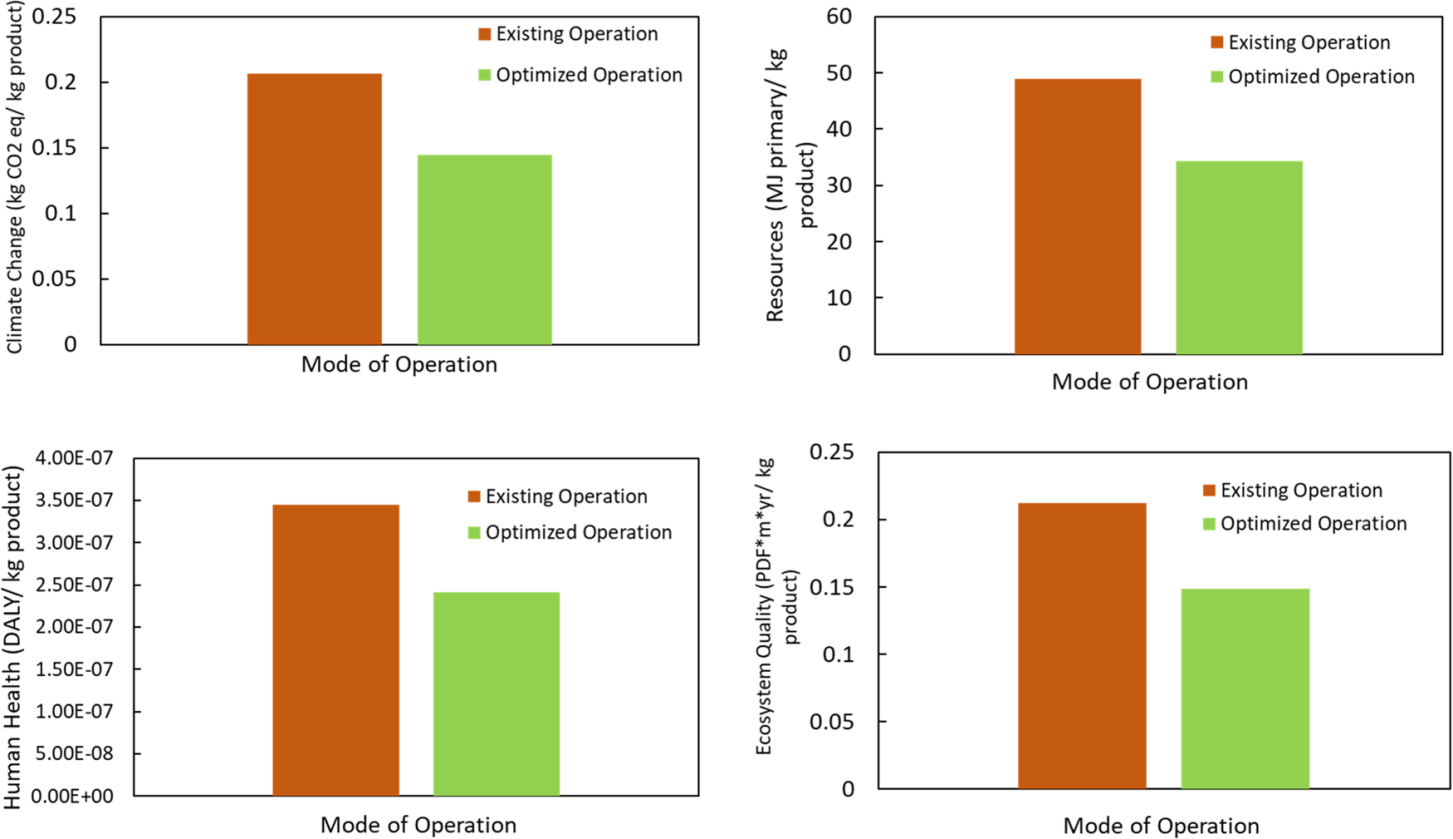
Life-cycle assessment for the existing mode of operation and optimized operation at the customized flow rate.

The existing trial and error method that is currently used at lubricant facilities often requires the flushing operation to be repeated for multiple iterations and, therefore, involves a significantly large associated flushing volume. Alternatively, if we apply the discretized NLP solution approach and conduct the operations at a customized flow rate, we can reduce the necessary flushing volume to over 30%. Figure 13 illustrates the required flushing volume in gallons for the existing mode of operation and the proposed customized flow rate via the discrete-time NLP solution approach. Thus, we believe that this approach has great potential to improve resource conservation and the environmental footprint of these operations. Life-cycle assessment (LCA) is a scientific method for systematically analyzing the environmental impact and the sustainability of various processes and products.^44−47^ We studied the life-cycle assessment of the optimized operation with the existing operation by using SimaPro software. We focus our calculations on the end-point assessment category considering human health (DALY), ecosystem quality (PDF m^2^ y), climate change (MT CO_2_-eq), and resource (MJ primary). The obtained results are illustrated in Figure 14.

## 6 Conclusions and Future Work

In this study, we confirmed the applicability of API-TDB-recommended viscosity blending correlations for lubricant mixtures by gathering experimental data from known compositions of lube oil blends. The correlations demonstrated close agreement with the experimental results, staying within a 5% margin of error. Additionally, we developed mathematical models based on first-principles to represent the flushing operations. These models were validated using experimental data obtained from a collaborative lubricant blending plant, with the validation showing an agreement within a 7% error margin. For the optimization of the flushing operation, we approached it as an optimal control problem and employed two solution methods: Pontryagin’s maximum principle and discrete-time nonlinear programming. While the maximum principle method had a considerably longer execution time, exceeding 70 000 s, and yielded unrealistic flushing time predictions, the discrete-time NLP solution approach completed in under 10 s and provided results that closely aligned with experimental data. Therefore, the discrete-time NLP solution approach holds significant potential for optimizing the trial-and-error-based flushing operation by minimizing the required flushing volume to meet the desired specifications.

Our research presents a valuable strategy for optimizing flushing times within the lubricant industry. This approach empowers operators to make well-informed decisions, reducing operational downtime and enhancing the economic, resource management, and environmental aspects of these processes.

Looking ahead, we plan to enhance our developed models by incorporating additional factors, such as viscosity, diffusion coefficients, and frictional losses, which are pivotal in fluid hydrodynamics. Additionally, we aim to explore the transferability of our solution approach to other sectors with similar operational procedures, including the specialty chemical industry, food production, personal care product manufacturing, and the pharmaceutical industry.

## Supporting Information Available

Additional details such as adjoint equations corresponding to the state equations, calculation of the Hamiltonian derivative, MATLAB code for solution using Pontryagin’s maximum principle, and MATLAB code for the solution to discretized-time nonlinear programming (PDF)
