# Supplementary material for: Strategic Optimization of the Flushing Operations in Lubricant Manufacturing and Packaging Facilities
Source: ACS Omega. 2023 Oct 7;8(41):38288–300. doi: 10.1021/acsomega.3c04668 (PMC10586454; doi:10.1021/acsomega.3c04668)
Supplement: Supplementary file 1 — ao3c04668_si_001.pdf [file ao3c04668_si_001.pdf]

Strategic Optimization of the Flushing Operations in Lubricants Manufacturing and Packaging Facilities

Swapana S. Jerpoth, Robert Hesketh, C. Stewart Slater, Mariano J. Savelski, Kirti M. Yenkie*
Department of Chemical Engineering, Rowan University, Glassboro, NJ 08028, USA

SUPPLEMENTARY MATERIAL

S1. Adjoint Equations Corresponding to the State Equations

$$\frac{dz_i}{dt} = - \sum_{j=1}^{n} z_j \frac{\partial f_j}{\partial x_i} \tag{S1}$$

$$\frac{dz_1}{dt} = z_1 \frac{Q_t}{A_C L} - z_2 \frac{Q_t}{A_C L} - z_3 [9{x_{A_t}}^2 \mu_A^{2/3} \frac{Q_t}{A_C L} (\mu_2^{1/3} - \mu_A^{1/3}) + (12x_{A_t} - 18{x_1}^2) \frac{Q_t}{A_C L} (\mu_A^{1/3} \mu_B^{2/3} - \mu_A^{2/3} \mu_B^{1/3}) + (3{x_{A_t}}^2 - 4x_{A_t} + 1) \frac{3Q}{A_C L} [\mu_B - \mu_A^{1/3} \mu_B^{2/3}] \tag{S2}$$

$$\frac{dz_2}{dt} = -z_1 \frac{Q_t}{A_C L} + z_2 \frac{Q_t}{A_C L} - z_3 [3\mu_A^{2/3} (-3{x_{B_t}}^2 + 6x_{B_t} - 3) \frac{Q_t}{A_C L} (\mu_B^{\frac{1}{3}} - \mu_A^{\frac{1}{3}}) + 6(1 - 4x_{B_t} + 3{x_{B_t}}^2) \frac{Q_t}{A_C L} (\mu_A^{\frac{1}{3}} \mu_B^{\frac{2}{3}} - \mu_A^{\frac{2}{3}} \mu_B^{\frac{1}{3}}) + 3\mu_B^{\frac{2}{3}} (2x_{B_t} - 3{x_{B_t}}^2) \frac{Q_t}{A_C L} (\mu_B^{\frac{1}{3}} - \mu_A^{\frac{1}{3}})] \tag{S3}$$

$$\frac{dz_3}{dt} = 0 \tag{S4}$$

S2. Calculation of the Hamiltonian Derivative

The maximum principle involves the maximization of Hamiltonian over the control variable, the optimality condition is [|dH/dQ_t |] < tolerance. The Hamiltonian is a function of several variables such as x, t, z, and Q_t, hence, its derivative can be expressed as a sum of partial derivatives, with respect to each of these variables t, x, y. The complete derivative with respect to one variable (t), is given by equation (S5).

$$\frac{dH}{dQ_t} = \sum_{i=1}^{3} \left(\frac{dH}{dx_i}\right)\left(\frac{dx_i}{dQ_t}\right) + \sum_{i=1}^{3} \left(\frac{dH}{dz_i}\right)\left(\frac{dz_i}{dQ_t}\right) \tag{S5}$$

$$where: \theta_i = \frac{dx_i}{dQ} \ \& \ \Phi_i = \frac{dz_i}{dQ_t} \tag{S6}$$

The following differential equations are to evaluate θ_i and Φ_i

$$\frac{d\left(\frac{dx_i}{dt}\right)}{dQ_t} = \frac{d\left(\frac{dx_i}{dQ_t}\right)}{dt} = \frac{d\theta_i}{dt} \tag{S7}$$

$$\frac{d\left(\frac{dz_i}{dt}\right)}{dQ_t} = \frac{d\left(\frac{dz_i}{dQ_t}\right)}{dt} = \frac{d\Phi_i}{dt} \tag{S8}$$

Thus, the general form of these two equations, in terms of the associated variables, can be written as

$$\frac{d\theta_i}{dt} = \mathrm{f}\,(x_i, Q_t, t) \tag{S9}$$

$$\frac{d\Phi_i}{dt} = \mathrm{f}\,(x_i, z_i, Q_t, t) \tag{S10}$$

For example, if we consider the first state equation for state variable x_A

$$\frac{d\theta_1}{dt} = \frac{-x_A}{A_C L} \tag{S11}$$

$$\frac{d\Phi_1}{dt} = \frac{-z_1 x_A}{A_C L} \tag{S12}$$

Equation (S9) is integrated in the forward direction, using a numerical method with the initial conditions of $\theta_i(t_0)$ = [0,0,0], while equation (S10) is integrated in the backward direction, with the final boundary conditions of $\Phi_i(t_f)$ = [0,0,0,0]

S3. MATLAB Code for Solution Using Pontryagin's Maximum Principle

S3.1 Deterministic Using Explicit Euler

```
clc
clear all
tic;
% ========= initial information Test Case 1 ==================================
del_t=0.05;
mu1=130.26;
mu2=305.39;
a=0.019;
l=6.375;
t0=0;     tf=290;  h=del_t;
time=t0:h:tf;
t_len=length(time);
%============initial conditions for state and theta==================
I0 = [1      0 130.26]';
Thi=[0 0 0]';
%============final conditions for adjoint and phi==================
Fz = [0 0 -1]';
Phif = [0 0 0]';
%Preallocation of space:
         x1=ones(1,t_len);
         x2=ones(1,t_len);
         x3=ones(1,t_len);

        alfa1 = ones(1,t_len);
        alfa2 = ones(1,t_len);
        alfa3 = ones(1,t_len);
% Preallocation of spaces for adjoints:
        z1= ones(1,t_len);
        z2= ones(1,t_len);
        z3= ones(1,t_len);
```

```
        zdot1=ones(1,t_len);
        zdot2=ones(1,t_len);
        zdot3=ones(1,t_len);

dtheta1 = ones(1,t_len);
dtheta2 = ones(1,t_len);
dtheta3 = ones(1,t_len);

 theta1 = ones(1,t_len);
 theta2 = ones(1,t_len);
 theta3 = ones(1,t_len);

        phi1 = ones(1,t_len);
        phi2 = ones(1,t_len);
        phi3 = ones(1,t_len);

dphi1= ones(1,t_len);
dphi2 = ones(1,t_len);
dphi3 = ones(1,t_len);
%======================initial guess for Q(flowrate)==================

Qflow = ones(1,t_len);
Qflownew = ones(1,t_len);
Qflow = 0.00303*Qflownew;

% Optimal flow
tolerance=1e-4*ones(1,t_len);
dHdQ = ones(1,t_len);
der1= ones(1,t_len);
der2 = ones(1,t_len);
H = ones(1,t_len);
numiter = 1;
p=1;
% while (abs(dHdT(:)) > tolerance(:))
for j=1:10
%================== solution of state equations========================
        x1 = 1;
        x2 = 0;
        x3 = 130.26;
  for i=1:(t_len)
        Q=Qflow(i);
        save Q

        alfa1(i) = -((x1(i))*(Q/(a*l)));
        alfa2(i) = (x1(i))*(Q/(a*l));
        alfa3(i)= (((x1(i))*(Q/(a*l)))*((3)*x3(i)^(2/3)))*((mu2^(1/3))-(mu1^(1/3)));

        x1(i+1) = x1(i) + alfa1(i)*del_t;
        x2(i+1) = x2(i) + alfa2(i)*del_t;
        x3(i+1) = x3(i) + alfa3(i)*del_t;
  end
     state = [x1' x2' x3'];
    saveallstate(:,p)=x1';
    saveallstate(:,p+1)=x2';
    saveallstate(:,p+2)= x3';
```

```
 p=p+4;
    figure(1)
    plot(time,Qflow); hold all
    ylabel ('Q_flow')
    xlabel ('Time')

%%Part II: the backward integration for adjoints:
%Final condition at time t = 290
Fz = [0 0 -1]';
%==================================================================
% Solution of adjoint equations: z equations
        zx1 = 0;
        zx2 = 0;
        zx3 = -1;
    for i=(t_len):-1:2
        Q=Qflow(i);
        save Q
        z1(t_len)=zx1;
        z2(t_len)= zx2;
        z3(t_len)= zx3;

zdot1(i)=(z1(i)*(Q/(a*l)))-(z2(i)*(Q/(a*l)))-
(z3(i)*((9*(mu1^(2/3))*(x1(i)^2)*(Q/(a*l))*((mu2^(1/3))-(mu1^(1/3))))+((12*x1(i)-
18*(x1(i)^2))*(Q/(a*l))*(((mu1^(1/3))*(mu2^(2/3)))-
((mu1^(2/3))*(mu2^(1/3)))))+((3*(Q/(a*l)))*(1-4*(x1(i))+3*(x1(i))^2))-((mu2)-
((mu1^(1/3))*(mu2^(2/3))))));

zdot2(i)=((-z1(i))*(Q/(a*l)))+(z2(i)*(Q/(a*l)))-(z3(i)*((-9*(mu1^(2/3))*((1-
x2(i))^2)*(Q/(a*l))*((mu2^(1/3))-(mu1^(1/3))))+(6*((1-
(4*x2(i))+(3*x2(i))^(2)))*(Q/(a*l))*(((mu1^(1/3))*(mu2^(2/3)))-
((mu1^(2/3))*(mu2^(1/3)))))+(3*(mu2^(2/3))*(2*(x2(i))-
3*(x2(i)^(2)))*(Q/(a*l))*((mu2^(1/3))-(mu1^(1/3))))));

zdot3(i)=0;

        z1(i-1) = z1(i) - zdot1(i)*del_t;
        z2(i-1) = z2(i) - zdot2(i)*del_t;
        z3(i-1) = z3(i) - zdot3(i)*del_t;

     end
     adjz = [z1' z2' z3' ];

%=======Solving for the Hamiltonian================================
%================== solution of theta equations========================
        th1 = 0;
        th2 = 0;
        th3 = 0;
  for i=1:(t_len)
        Q=Qflow(i);
        save Q

        theta1(1)= th1;
        theta2(1)= th2;
        theta3(1)= th3;
```

```
dtheta1(i) = -x1(i)/(a*l);
dtheta2(i)=x1(i)/(a*l);
dtheta3(i) = (3*(mu1^(2/3))*(x1(i)^3)*(1/(a*l))*((mu2^(1/3))-
(mu1^(1/3))))+(6*(x1(i)^2)*x2(i)*(1/(a*l))*(((mu1^(1/3))*(mu2^(2/3)))-
((mu1^(2/3))*(mu2^(1/3)))))+(3*(mu2^(2/3))*x1(i)*(x2(i)^2)*(1/(a*l))*((mu2^(1/3))-
(mu1^(1/3))));

 theta1(i+1) = theta1(i) + dtheta1(i)*del_t;
 theta2(i+1) = theta2(i) + dtheta2(i)*del_t;
 theta3(i+1) = theta3(i) + dtheta3(i)*del_t;

 J(i)= ((x3(i)-mu2)^2);
 Objective=J(i);
 end
  sol_Theta= [theta1' theta2' theta3'];

% ===================solution of phi equations==========================
   sol_Phi=zeros(t_len,3);
   phi=Phif';
        phix1 = 0;
        phix2 = 0;
        phix3 = 0;
    for i=(t_len):-1:2
        Q=Qflow(i);
        save Q
        phi1(t_len)=phix1;
        phi2(t_len)= phix2;
        phi3(t_len)= phix3;

dphi1(i)= -z1(i)*(x1(i)/(a*l));
dphi2(i) = z2(i)*(x1(i)/(a*l));
dphi3(i) = z3(i)*((3*(mu1^(2/3))*(x1(i)^3)*(1/(a*l))*((mu2^(1/3))-
(mu1^(1/3))))+(6*(x1(i)^2)*(x2(i))*((1/(a*l)))*(((mu1^(1/3))*(mu2^(2/3)))-
((mu1^(2/3))*(mu2^(1/3)))))+(3*(mu2^(2/3))*(x1(i))*(x2(i)^2)*((1/(a*l)))*(((mu2^(1/3)
)-(mu1^(1/3))))));

        phi1(i-1) = phi1(i) - dphi1(i)*del_t;
        phi2(i-1) = phi2(i) - dphi2(i)*del_t;
        phi3(i-1) = phi3(i) - dphi3(i)*del_t;
     end
     sol_Phi = [phi1' phi2' phi3'];

%====================Hamiltonian%evaluation=========
    dHdx=zeros(t_len,3);
    dHdz=zeros(t_len,3);
for i=1:(t_len)
    Q=Qflow(i);
    save Q
    Xs=[state(i,1) state(i,2) state(i,3)]';
    Zs=[adjz(i,1) adjz(i,2) adjz(i,3)]';
    dHdx(i,:) =feval(@derHdX_pipe_flushing,Xs,Zs);
end
for i=1:(t_len)
    Q=Qflow(i);
    save Q
```

```
    Xs =[state(i,1) state(i,2) state(i,3) ]';
    dHdz(i,:) =feval(@derHdZ_pipe_flushing,Xs);
end
    for i=1:(t_len)
        der1(i)=(dHdx(i,1)*theta1(i)+dHdx(i,2)*theta2(i)+dHdx(i,3)*theta3(i));
        der2(i)=(dHdz(i,1)*phi1(i)+dHdz(i,2)*phi2(i)+dHdz(i,3)*phi3(i));
        dHdQ(i)= der1(i) + der2(i);
        H(i)= z1(i)*dHdz(i,1)+z2(i)*dHdz(i,2)+z3(i)*dHdz(i,3);
    end

figure(2)
plot(time,(H)); hold all
ylabel ('Hamiltonian')
xlabel ('Time')

figure(3)
plot(time,(dHdQ)); hold all
ylabel ('dHdQ')
xlabel ('Time')

    for k=1:t_len
        updt = 1e-6;
        Qflownew(k)=Qflow(k) + updt*dHdQ(k);
        Q_iters(k)= Qflownew(k);
        Qflow(k)=Qflownew(k);

    if (Qflow(k)<0)
        Qflow(k)=0;
    else if (Qflow(k)>0)
        Qflow(k)=Qflow(k);
    end
    end
    end

    for i=1:t_len
    der_H(j,i) = dHdQ(i);
    Flowrate(j,i) = Qflow(i);
    Hamiltonian(j,i) = H(i);
    end
    numiter = numiter+1;
end
    toc;

figure(4)
plot(time, Qflow)
ylabel ('Flowrate profile')
xlabel ('Time')
```

S3.2 Hamiltonian Equations (Derivative of Hamiltonian with respect to state variables)

```
function D1=derHdX_pipe_flushing(x,z)

load Q
mu1=130.26;
mu2=305.39;
a=0.019;
l=6.375;
dHdx(1)=z(1)*(-
Q/(a*l))+z(2)*(Q/(a*l))+z(3)*((9*(mu1^(2/3))*(x(1)^2)*(Q/(a*l))*((mu2^(1/3))-
(mu1^(1/3))))+(6*(2*(x(1))-(3*(x(1)^2))*(Q/(a*l))*(((mu1^(1/3))*(mu2^(2/3)))-
((mu1^(2/3))*(mu2^(1/3))))))+(3*(mu2^(2/3))*((mu2^(1/3))-(mu1^(1/3)))*((Q/(a*l)))*(1-
(4*x(1))+(3*(x(1)^2)))));
dHdx(2)=z(1)*(Q/(a*l))-z(2)*(Q/(a*l))+z(3)*((-9*(mu1^(2/3))*((1-
x(2))^2)*(Q/(a*l))*((mu2^(1/3))-(mu1^(1/3))))+(6*(1-
(4*x(2))+(3*(x(2)^2)))*(Q/(a*l))*(((mu1^(1/3))*(mu2^(2/3)))-
((mu1^(2/3))*(mu2^(1/3)))))+(3*(mu2^(2/3))*((mu2^(1/3))-
(mu1^(1/3)))*(Q/(a*l))*(2*x(2)-(3*(x(2)^2)))));
dHdx(3)=0;
D1=[dHdx(1) dHdx(2) dHdx(3)]';
```

S3.3 Hamiltonian Equations (Derivative of Hamiltonian with respect to adjoint variables)

```
function D2=derHdZ_pipe_flushing(x)

load Q
mu1=130.26;
mu2=305.39;
a=0.019;
l=6.375;
dHdz(1) = -(x(1)*(Q/(a*l)));
dHdz(2) = (x(1)*(Q/(a*l)));
dHdz(3) = (3*(mu1^(2/3))*(x(1)^3)*(Q/(a*l))*((mu2^(1/3))-
(mu1^(1/3))))+(6*(x(1)^2)*x(2)*(Q/(a*l))*(((mu1^(1/3))*(mu2^(2/3)))-
((mu1^(2/3))*(mu2^(1/3)))))+ (3*(mu2^(2/3))*x(1)*(x(2)^2)*(Q/(a*l))*((mu2^(1/3)-
(mu1^(1/3)))));
D2=[dHdz(1) dHdz(2) dHdz(3)]';
```

S4. MATLAB Code for Solution Using Discrete-Time Non-Linear Programming

S4.1 Main File

```
function Q = mult_obj_optflush_v2(q)
%Parameter values (Test Case 1)
mu1=130.26;
mu2=305.39;
a=0.019; %cross sectional area
l=6.375; %pipe length
% Q=0.007;
Tinitial=0;
Tfinal=290;
```

```
h=1;
N = (Tfinal - Tinitial)/h;
t = [Tinitial:h:Tfinal]';
sum1 =0;
% Model equations:
for i=1:1:length(t)

%----------- integrated form of equations --------------%

x1(i) = (exp(-q(i)*t(i)/(a*l)));
x2(i) = 1-(exp(-q(i)*t(i)/(a*l)));
x3(i) = [(x1(i)*(mu1^(1/3)))+(x2(i)*(mu2^(1/3)))]^3;
obj(i)=((x3(i)-(mu2))/mu2)^2; %x3 is the mixture viscosity
sum1=sum1+obj(i);
end
%objective function:
% sum1
Q=sum1;
end
```

S4.2 Solver File

```
function [x,fval,exitflag,output,lambda,grad,hessian] = opt_fmin_solver_v2(x0,lb,ub)
x0=[0.005*ones(291,1);]; %initial guess for decision variables
lb=[0*ones(291,1);];
ub=[0.00303*ones(291,1);];
% Start with the default options
options = optimset;
% Modify options setting
options = optimset(options,'Display' ,'iter');
options = optimset(options,'PlotFcns' ,{  @optimplotx @optimplotfval });
options = optimset(options,'Diagnostics' ,'on');
options = optimset(options,'LargeScale' ,'on');
options = optimset(options,'TolX',1e-35,'TolFun',1e-35,'TolCon',1e-35);
[x,fval,exitflag,output,lambda,grad,hessian] =
fmincon(@mult_obj_optflush_v2,x0,[],[],[],[],lb,ub,[],options);
save output_mult_obj_optflush
end
```

S5. GitHub Link to Codes

https://github.com/kmygroup/Lubricant-Packaging.git
